# Predictors of Hospital Admission Following Emergency Department Presentation for Falls Among Older Adults: A Retrospective Analysis

**DOI:** 10.1111/ajag.70157

**Published:** 2026-04-05

**Authors:** Yong‐Joon Kim, Hyo‐Seon Shin, Kyeongmin Jang

**Affiliations:** ^1^ Department of Emergency Medicine Seoul National University–Seoul Metropolitan Government Boramae Medical Center Seoul Republic of Korea; ^2^ Department of Emergency Medical Services Sun Moon University Chungcheongnam‐do Republic of Korea; ^3^ Department of Nursing College of Health Sciences, Daejin University Gyeonggi‐do Republic of Korea

**Keywords:** accidental falls, aged, emergency service, hospital, hospitalisation, risk factors

## Abstract

**Objective:**

To identify demographic, clinical and injury‐related factors independently associated with hospital admission among older adults presenting to the emergency department (ED) after a fall.

**Methods:**

We conducted a retrospective chart review of electronic medical records for 574 patients aged 65 years and older who presented to the ED of a general hospital between January and June 2023 with non‐traumatic fall presentations. Data included demographics, fall circumstances, mode of arrival, comorbidities, medication exposure and ED diagnoses. Multivariable logistic regression was used to identify independent predictors of hospital admission.

**Results:**

Of 574 older adults, 187 (33%) were admitted. Admission was independently associated with female sex (aOR 2.29, 95% CI 1.35–3.89), age 78 years and older and ambulance arrival. Comorbidities including hypertension, diabetes, dementia, Parkinson's disease and multimorbidity were also associated with higher odds of admission. Use of fall‐risk‐increasing medications remained a significant predictor. Serious injuries, particularly fractures and intracranial haemorrhage, were strongly associated with admission.

**Conclusions:**

Hospital admission after ED presentation for falls in older adults reflects both injury severity and underlying clinical vulnerability. Incorporating comorbidity burden and medication risk into ED assessment may support disposition decisions and help target secondary prevention, especially for individuals discharged directly from the ED.

## Impact Statements

The findings may assist emergency clinicians in early risk stratification, disposition planning, support medication review and targeted secondary prevention, particularly for older adults discharged directly from the emergency department (ED). At a service level, these results can inform the design of ED‐to‐community care pathways aimed at reducing preventable admissions and improving outcomes.

## 1 Introduction

Falls are a major public health concern among older adults, contributing to substantial morbidity, mortality and healthcare expenditure [1]. With global population ageing, fall‐related emergency department (ED) presentations and hospitalisations are steadily increasing, posing a significant challenge to health systems [2]. In this context, identifying factors associated with hospital admission following ED presentation for a fall is clinically relevant because admission decisions determine not only acute management but also downstream care pathways and resource allocation.

The ED is a critical point of contact after a fall. Clinicians must rapidly determine whether hospital admission is warranted, balancing injury severity with medical complexity, monitoring needs and discharge safety. Importantly, older adults discharged directly from the ED may not receive structured follow‐up despite being at risk of functional decline and recurrent falls, highlighting the ED as a potential window of opportunity for targeted secondary prevention [3].

Prior evidence has examined demographic, environmental and clinical contributors to admission risk; however, findings have not always been consistent across settings and study designs. For example, sex differences in admission rates vary across studies, potentially reflecting differences in injury patterns, frailty and fracture susceptibility [4, 5]. Advancing age is consistently associated with hospitalisation, likely reflecting reduced physiological reserve and higher comorbidity burden [6]. Environmental and situational characteristics—including indoor versus outdoor falls and prehospital transport by ambulance—may further shape admission decisions by signalling frailty, urgency or perceived injury severity [7, 8].

Comorbidity burden and medication exposure are also clinically important, yet their relative contribution may be difficult to disentangle when considered in isolation. Hypertension, diabetes, dementia, osteoporosis and Parkinson's disease have been linked to adverse outcomes following falls [9, 10], and potentially inappropriate medications (PIMs) may increase vulnerability through sedation, orthostatic effects or impaired balance [11]. Evidence regarding polypharmacy as an independent risk factor remains inconclusive [12], and social context (e.g., living arrangement and availability of support) may influence discharge safety and admission decisions [13]. These considerations suggest that a multidimensional approach that evaluates demographic, situational, clinical and medication‐related factors simultaneously may better reflect real‐world ED decision‐making.

Accordingly, this study aimed to identify factors associated with hospital admission among older adults presenting to the ED following a fall, to inform clinical risk assessment and support care planning for both admitted and discharged patients in geriatric emergency care. Recent work highlighting the complexity of care needs after ED discharge further underscores the importance of clarifying admission‐related risk profiles in this setting [14].

## 2 Methods

### 2.1 Study Design and Population

This retrospective observational study examined electronic medical records (EMRs) of adults aged 65 years and older who presented to the ED of a general hospital in South Korea for a fall‐related visit between January and June 2023.

In this ED, the EMR routinely categorises the presenting reason as traumatic or non‐traumatic, enabling systematic identification of fall presentations driven primarily by medical or functional factors. We focused on non‐traumatic fall presentations (e.g., dizziness, gait instability or functional decline) because admission decisions in this group are more strongly influenced by clinical complexity, comorbidities, monitoring needs and discharge safety. In contrast, traumatic or injury‐mechanism falls are often admitted due to injury severity itself, which can dominate admission decisions and obscure associations with patient‐level predictors.

**FIGURE 1:**
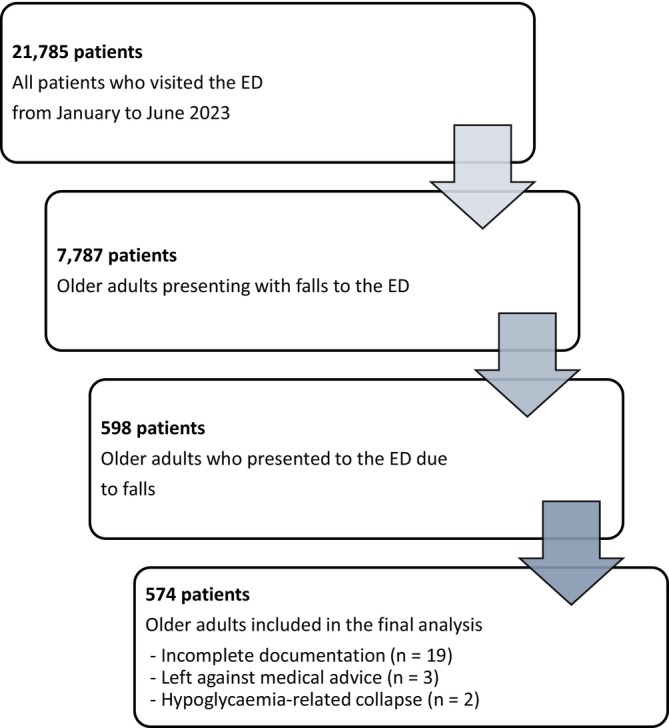
Flowchart of patient selection for analysis of older adult falls in the emergency department (ED).

A fall was defined according to the World Health Organization as an event in which a person comes to rest inadvertently on the ground, floor or a lower level. Cases were reviewed at the individual‐record level to confirm eligibility based on ED physician documentation and clinical notes. Presentations primarily attributable to syncope, intentional injury or acute medical events not related to balance or gait were not included in the analytic cohort to improve interpretability and cohort homogeneity. The detailed flow of inclusions and exclusions is reported in Figure 1.

Hospital admission decisions were made by the attending ED physicians based on clinical assessment, including injury findings, need for inpatient management of comorbidities, requirement for monitoring and considerations related to discharge safety and available social support.

### 2.2 Data Collection and Study Variables

Data were retrospectively extracted from the hospital's EMR system using a standardised abstraction form. Variables included demographics, fall circumstances, mode of ED arrival, comorbidities, medication exposure and ED discharge diagnoses.

Demographics comprised age, sex, living arrangement (alone, with family or residential facility), body mass index (BMI) and insurance type (National Health Insurance vs. Medical Aid). Age was analysed as a continuous variable and, for selected analyses, dichotomised using an optimal cut‐off derived from receiver operating characteristic (ROC) curve analysis for hospital admission. Body mass index was analysed as a continuous variable in regression models and categorised for descriptive comparisons as underweight (< 18.5 kg/m^2^), normal (18.5–24.9 kg/m^2^) or overweight (≥ 25 kg/m^2^).

Fall‐related characteristics included fall location (indoors vs. outdoors), mechanism (slip, trip or fall from height) and mode of arrival (ambulance, private vehicle or ambulatory).

Comorbidities were identified from documented medical history and included hypertension, diabetes mellitus, osteoporosis, dementia, Parkinson's disease and cerebrovascular disease. Multimorbidity was defined as the presence of two or more chronic conditions recorded in the medical history.

Medication exposure was determined from the active medication list at the time of the ED visit. Polypharmacy was defined as concurrent use of five or more medications. Potentially inappropriate medications (PIMs) were identified using the 2023 American Geriatrics Society Beers Criteria. This approach was chosen because it is widely used and could be applied consistently to EMR medication data; however, fall‐specific tools (e.g., STOPP‐Fall) may provide greater specificity for fall‐risk‐increasing drugs.

Emergency department discharge diagnoses were grouped into clinically relevant injury categories: limb fractures, torso or facial fractures, intracranial haemorrhage (confirmed by imaging, such as CT or MRI) and contusions or lacerations.

The primary outcome was hospital admission following the ED visit, defined by ED disposition (admitted vs. discharged), reflecting clinical judgements regarding injury findings, medical management needs, monitoring requirements and discharge safety.

### 2.3 Statistical Analysis

All analyses were conducted using IBM SPSS Statistics for Windows, Version 29.0 (IBM Corp., Armonk, NY, USA). Descriptive statistics are presented as *n* (%) for categorical variables and mean (SD) for continuous variables.

Group differences were examined using the *χ* ^2^ test for categorical variables and the independent‐samples *t*‐test for continuous variables. Crude associations with hospital admission were expressed as unadjusted odds ratios (ORs) with 95% confidence intervals (CIs). Bonferroni‐adjusted pairwise comparisons were applied for multi‐level categorical variables when appropriate.

An age cut‐off for hospital admission was derived using ROC curve analysis, with the optimal threshold selected by the Youden index. Multivariable binary logistic regression was then performed to identify independent predictors of admission. Candidate variables were selected based on clinical relevance and/or univariable associations and included demographics, fall characteristics, mode of arrival, comorbidities, multimorbidity, polypharmacy, PIM use and ED discharge diagnosis categories. Adjusted odds ratios (aORs) with 95% CIs are reported. Multicollinearity was assessed using variance inflation factors (VIFs).

Model calibration was evaluated with the Hosmer–Lemeshow test, and performance was summarised using Nagelkerke's R^2^ and classification indices (overall accuracy, sensitivity, specificity). Statistical significance was set at *p* < 0.05.

### 2.4 Ethical Considerations

This study was approved by the Institutional Review Board of Seoul Metropolitan Government–Seoul National University Boramae Medical Center (IRB No.: 30–2024‐85) and conducted in accordance with the ethical principles of the Declaration of Helsinki. Given the retrospective nature of the study, a waiver of informed consent was granted by the ethics committee.

To protect participant anonymity, all data were de‐identified prior to analysis. No personally identifiable information was collected or retained. Only anonymised, aggregate data were used in statistical analyses, and all results were reported in a way that precludes the identification of individual participants.

## 3 Results

### 3.1 Study Sample

During the study period, 21,785 individuals visited the ED, of whom 7787 were aged 65 years or older. Among these, 598 presented with fall‐related complaints. After individual‐record review, 24 cases were excluded due to incomplete documentation (*n* = 19), leaving against medical advice (*n* = 3) or hypoglycaemia‐related collapse (*n* = 2). The final analytic sample comprised 574 older adults with non‐traumatic fall presentations (Figure 1).

### 3.2 Predictors of Hospital Admission: Multivariable Logistic Regression

Multivariable logistic regression analysis identified several independent predictors of hospital admission among older adults presenting to the ED after a fall (Table 1). Female sex was associated with significantly higher odds of admission compared with males (aOR 2.29, 95% CI 1.35–3.89). Older age (78 years and older) was also independently associated with admission.

**TABLE 1:** Binary logistic regression analysis of factors associated with hospitalisation among older adults.

Variables	aOR	*p*	95% CI for Exp(B) (Lower–upper)
Sex (female vs. male)	2.288	0.002	1.347–3.887
Living situation			
Living family (vs. alone)	1.154	0.53	0.644–2.069
Nursing home (vs. alone)	0.274	0.21	0.041–1.822
Age ≥ 78 years (vs < 78 years)	1.911	0.03	1.151–2.133
BMI (per 1 kg/m^2^ increase)	0.792	0.43	0.610–1.234
Medical aid (vs. National Health Insurance)	0.492	0.07	0.227–1.062
Indoor fall (vs. outdoor fall)	1.056	0.83	0.648–1.719
Cerebrovascular disease (yes vs. no)	1.208	0.63	0.566–2.581
Hypertension (yes vs. no)	2.671	0.01	1.247–5.721
Diabetes (yes vs. no)	1.948	0.03	1.060–3.580
Osteoporosis (yes vs. no)	0.883	0.68	0.489–1.596
Dementia (yes vs. no)	1.659	0.03	1.121–1.911
Parkinson's disease (yes vs. no)	6.182	0.02	1.399–27.315
Multiple chronic conditions (yes vs. no)	3.058	0.002	1.511–6.211
Use of five or more medications (yes vs. no)	0.301	0.09	0.081–1.212
Fall‐risk‐increasing medication use (yes vs. no)	3.861	< 0.001	1.905–7.813
Mechanism of injury			
Tripping (vs slipping)	0.732	0.61	0.532–1.417
Falling from height (vs slipping)	1.378	0.46	0.512–3.919
Mode of arrival		< 0.001	
Ambulance (vs private vehicle)	6.501	0.01	1.543–26.244
Walk‐in (vs private vehicle)	0.499	< 0.001	0.308–0.811
Constant	1.868	0.68	

*Note:* Model fit: Hosmer–Lemeshow test indicated good fit (*χ* ^2^ = 3.262, *p* = 0.92). Model explanatory power: Nagelkerke's *R* ^2^ = 0.316 (32% of variance explained). Classification accuracy: overall accuracy = 71%, specificity = 81%, sensitivity = 56%. Results are reported as adjusted odds ratios (aORs) with 95% confidence intervals (CIs); statistical significance set at *p* < 0.05.

Abbreviations: aOR, adjusted odds ratio; BMI, body mass index; CI, confidence interval.

Clinical comorbidities demonstrated strong associations with hospitalisation. Hypertension, diabetes mellitus, dementia and Parkinson's disease were each independently related to increased admission risk. In addition, multimorbidity (two or more chronic conditions) more than tripled the likelihood of admission, and the use of fall‐risk‐increasing medications was similarly associated with substantially higher odds.

Prehospital factors were also important. Arrival by ambulance markedly increased the probability of admission, whereas walk‐in presentation was associated with lower odds. In contrast, BMI, insurance type, fall location, cerebrovascular disease, osteoporosis, polypharmacy and injury mechanism were not independently associated with admission after adjustment.

The model demonstrated good calibration (Hosmer–Lemeshow *p* = 0.92) and moderate explanatory power (Nagelkerke's *R* ^2^ = 0.316), with an overall classification accuracy of 71%.

### 3.3 Baseline Demographic Characteristics

Baseline demographic characteristics of the study population are presented in Table 2. Of the 574 older adults, 187 (33%) were admitted to hospital. Admitted participants were older on average, and a higher proportion were female compared with those discharged from the ED. Living arrangement also differed between groups, with individuals living with family more frequently hospitalised than those living alone.

**TABLE 2:** General characteristics of hospitalised and non‐hospitalised older adults who visited the emergency department due to falls.

Variables	Hospitalised (*n* = 187)	Non‐hospitalised (*n* = 387)	*χ* ^2^ or *t*	*p*
Sex (male, %)	48 (26)	184 (48)	25.056	< 0.001
Age (years, Mean ± SD)	78.71 (7.785)	75.55 (7.533)	−3.182	0.002
Living situation, *n* (%)				
Living alone	42 (23)	126 (33)	6.36	0.04
Living with family	141 (75)	252 (65)		
Nursing home	4 (2)	9 (2)		
BMI (kg/m^2^, Mean ± SD)				
Underweight (< 18.5)	19 (10.2)	23 (5.9)	42.84	< 0.001
Normal (18.5–24.9)	61 (32.6)	78 (20.2)		
Overweight (≥ 25)	80 (42.8)	126 (32.6)		
Insurance type, *n* (%)				
National Health Insurance	169 (90)	333 (86)	2.153	0.09
Medicaid	18 (10)	54 (14)		

*Note:* Values are presented as number (percentage) or mean ± standard deviation (SD). Pairwise comparisons performed using Bonferroni correction after significant *χ* ^2^ tests. Living Situation: ‘Living with Family’ significantly differed from ‘Living Alone’ (b > a, *p* = 0.047); ‘Nursing Home’ not significantly different from others. BMI: Although the overall *χ* ^2^ test showed a significant difference among three BMI categories (*p* < 0.001), no specific significant differences were found in pairwise comparisons after Bonferroni correction.

Abbreviations: BMI, body mass index; χ^2^, chi‐square test; *t*, independent *t*‐test.

The distribution of body mass index categories varied across groups; however, pairwise comparisons did not demonstrate significant differences after adjustment for multiple testing. Insurance type showed no significant association with hospital admission.

### 3.4 Fall‐Related Characteristics and Injury Outcomes

Fall‐related circumstances and injury patterns differed between participants who were admitted and those discharged (Table 3). Indoor falls were more common among admitted individuals, whereas outdoor falls were more frequently observed among those discharged. The mechanism of injury (slip, trip or fall from height) did not significantly differ between groups.

**TABLE 3:** Fall‐related characteristics and final diagnoses of hospitalised and non‐hospitalised older adults who visited the emergency department.

Variables	Hospitalised (*n* = 187)	Non‐hospitalised (*N* = 387)	*χ* ^2^ or *t*	*p*
Fall location, *n* (%)				
Indoor	107 (57)	172 (44)	8.237	0.003
Outdoor	80 (43)	215 (56)		
Mechanism of injury, *n* (%)				
Slipping	116 (62)	224 (58)	1.209	0.54
Tripping	58 (31)	138 (36)		
Falling from height	13 (7)	25 (7)		
Mode of arrival, *n* (%)				
Ambulance	117 (63)	185 (48)	29.33	< 0.001
Private vehicle	44 (24)	102 (26)		
Walking	26 (14)	100 (26)		
Final diagnosis, *n* (%)				
Fracture (limb)	138 (74)	24 (6)	391.675	< 0.001
Fracture (torso/face)	15 (8)	39 (10)		
Brain haemorrhage	24 (13)	1 (0)		
Contusion and laceration	10 (5)	323 (84)		

*Note:* Values are presented as number (percentage). *χ* ^2^ = chi‐square test. Pairwise comparisons were performed using Bonferroni correction after significant *χ* ^2^ tests. Mode of Arrival: ‘Ambulance’ significantly differed from ‘Walking’ (Ambulance > Walking, *p* = 0.001); ‘Private vehicle’ was not significantly different from either group. Final diagnosis: ‘Fracture (limb)’ and ‘Brain haemorrhage’ groups showed significantly higher hospitalisation rates compared with ‘Fracture (torso/face)’ and ‘Contusion & laceration’. ‘Fracture (torso/face)’ also showed significantly higher hospitalisation compared with ‘Contusion & laceration’ (Fracture (limb) = Brain haemorrhage > Fracture (torso/face) > Contusion and laceration, all *p* < 0.001, except limb vs. haemorrhage).

Mode of arrival showed a clear association with hospitalisation. Participants transported by ambulance were substantially more likely to be admitted, whereas walk‐in presentations were more common among those discharged.

Marked differences were observed in injury severity. Fractures, particularly limb fractures, and intracranial haemorrhage occurred predominantly among admitted participants, whereas minor injuries such as contusions or lacerations were mainly managed without hospitalisation.

### 3.5 Comorbidities and Medication Use

Several clinical characteristics differed between admitted and non‐admitted participants (Table 4). Admitted individuals more frequently had chronic conditions, including hypertension, osteoporosis, dementia and Parkinson's disease, and a greater proportion met criteria for multimorbidity (two or more chronic conditions).

**TABLE 4:** Comorbidities and medication use among study subjects.

Variables	Hospitalised (*n* = 187)	Non‐hospitalised (*N* = 387)	χ^2^ or *t*	*p*
Cerebrovascular disease, *n* (%)	20 (11)	29 (8)	1.655	0.13
Hypertension, *n* (%)	115 (62)	187 (48)	4.908	0.02
Diabetes, *n* (%)	58 (31)	119 (31)	0.004	0.51
Osteoporosis, *n* (%)	51 (27)	46 (12)	21.254	< 0.001
Dementia, *n* (%)	18 (10)	11 (3)	12.093	< 0.001
Parkinson's disease, *n* (%)	21 (11)	15 (4)	4.142	0.002
Multiple chronic conditions (two or more), *n* (%)	95 (51)	100 (26)	35.024	< 0.001
Total number of medications (five or more), *n* (%)	11 (6)	19 (5)	3.132	0.09
Fall‐inducing medication use, *n* (%)	105 (56)	112 (29)	39.698	< 0.001

*Note:* Values are presented as number (percentage). *χ* ^2^ = chi‐square test. No pairwise comparisons were necessary as all variables had only two categories (binary variables).

Medication‐related factors also differed between groups. The use of fall‐risk‐increasing medications was more common among admitted participants, whereas the prevalence of polypharmacy did not significantly differ between groups.

## 4 Discussion

This study identified factors independently associated with hospital admission among older adults presenting to the ED following a fall. The main added value of this work is the use of a multidimensional model that jointly considered demographic characteristics, clinical vulnerability (comorbidities and multimorbidity), medication exposure and presentation features, thereby reflecting the complexity of ED disposition decisions rather than relying on isolated univariable comparisons.

Female sex and older age (78 years and older) were associated with increased likelihood of hospitalisation. Higher admission rates among older women have been reported previously and may be related to frailty and fracture susceptibility, including osteoporosis‐related fractures [5]. However, other studies have found higher admission rates among men, potentially due to greater injury severity [4]. These discrepancies likely reflect differences in case‐mix, injury classification and admission thresholds across health systems. Advanced age remains a consistent predictor of admission, plausibly reflecting reduced physiological reserve, slower recovery and greater chronic disease burden after falls [6, 15, 16].

A key finding was that clinical vulnerability indicators—hypertension, diabetes, dementia, Parkinson's disease and multimorbidity—were independently associated with admission, supporting prior evidence that underlying medical complexity influences ED disposition decisions [17, 18]. The particularly strong association for Parkinson's disease is clinically plausible given motor impairment, postural instability and higher risk of serious injury and care needs [18]. Because cognitive status was captured only through documented diagnoses, subtle cognitive impairment could not be assessed; recent ED‐based evidence suggests that probable mild cognitive impairment may be linked with higher recurrent‐fall risk and functional consequences among non‐hospitalised older adults after severe falls [19].

Regarding fall circumstances, unadjusted differences (e.g., indoor vs. outdoor) should be interpreted cautiously. In our adjusted model, fall location did not remain independently associated with admission, suggesting that the apparent relationship may be explained by correlated vulnerability factors (e.g., comorbidity burden, cognitive/motor impairment and medication exposure) [20, 21]. In contrast, ambulance transport was independently associated with admission, consistent with prior work indicating that ambulance arrival can act as a proxy for clinical urgency, injury severity and discharge safety concerns in ED practice [22, 23]. This supports the practical use of arrival mode as an early cue for heightened assessment and disposition planning.

Medication exposure also has direct clinical implications. Potentially inappropriate medications identified using the 2023 Beers Criteria were associated with hospitalisation, consistent with evidence linking sedatives, antihypertensives, antipsychotics and benzodiazepines with impaired balance and adverse outcomes [11, 24]. However, Beers is a general PIM tool rather than fall‐specific; fall‐focused instruments such as the STOPP‐Fall list and deprescribing guidance may provide greater specificity for fall‐risk‐increasing drugs and could improve the clinical interpretability of medication‐related findings [25]. Polypharmacy was not independently associated with admission, suggesting that the medication risk profile may be more informative than medication count alone in this ED population [12].

As expected, injury type remained closely linked to admission. Limb fractures and intracranial haemorrhage were strongly associated with hospitalisation, reflecting the need for orthopaedic/surgical or neurological management and monitoring [18, 26, 27]. Importantly, older adults presenting to the ED after a fall can be broadly divided into those admitted and those discharged directly from the ED; these groups may require different follow‐up strategies. Recent ED‐based studies highlight both the feasibility and challenges of initiating falls prevention pathways at the ED interface, particularly for discharged patients who may otherwise lack structured follow‐up [28].

Several limitations warrant consideration. The single‐centre retrospective design may limit generalisability, and reliance on medical record documentation may have introduced misclassification and under‐ascertainment of geriatric syndromes (e.g., frailty, subtle cognitive impairment) and social risk factors. In addition, recurrent falls could not be reliably evaluated if prior fall history was inconsistently documented; future research should explicitly capture recurrent falls and repeat ED presentations. Finally, the absence of standardised frailty and injury severity measures constrains interpretation of clinical complexity influencing admission decisions. Prospective multicentre studies incorporating structured frailty/cognition screening, fall‐specific medication tools and post‐discharge follow‐up are needed to strengthen risk stratification and guide ED care pathways.

## 5 Conclusions

This study identified multiple factors independently associated with hospitalisation among older adults presenting to the ED following a fall. Female sex, advanced age (78 years and older), indoor falls, ambulance transport, comorbidities (including hypertension, diabetes, dementia and Parkinson's disease), multiple chronic conditions, use of PIMs and serious injuries such as fractures and brain haemorrhage were all linked to increased likelihood of admission.

These findings underscore the need for targeted fall‐prevention strategies, particularly for older adults with chronic illnesses or high‐risk medication profiles. Proactive risk screening, regular medication reviews and evidence‐based triage protocols in the ED may help reduce preventable admissions and improve care efficiency.

Future research should incorporate standardised frailty and injury severity assessments, and adopt longitudinal designs to examine post‐discharge outcomes. Such efforts are critical for refining risk stratification, guiding clinical decision‐making and enhancing the quality of care for older adults affected by falls.

## Funding

This research received no specific grant from any funding agency in the public, commercial or not‐for‐profit sectors.

## Ethics Statement

This study was approved by the Institutional Review Board of Seoul Metropolitan Government–Seoul National University Boramae Medical Center (IRB No.: 30–2024‐85). All procedures were conducted in accordance with the Declaration of Helsinki.

## Conflicts of Interest

The authors declare no conflicts of interest.

## Data Availability

The data that support the findings of this study are available on request from the corresponding author. The data are not publicly available due to privacy or ethical restrictions.
